# Kupffer cells-dependent inflammation in the injured liver increases recruitment of mesenchymal stem cells in aging mice

**DOI:** 10.18632/oncotarget.6744

**Published:** 2015-12-23

**Authors:** Xue Yang, Lei Liang, Chen Zong, Fobao Lai, Pengxi Zhu, Yu Liu, Jinghua Jiang, Yang Yang, Lu Gao, Fei Ye, Qiudong Zhao, Rong Li, Zhipeng Han, Lixin Wei

**Affiliations:** ^1^ Tumor Immunology and Gene Therapy Center, Eastern Hepatobiliary Surgery Hospital, the Second Military Medical University, Shanghai, China; ^2^ Medical College of Soochow University, Suzhou, China; ^3^ Department of Pharmacy, Chang Hai Hospital, the Second Military Medical University, Shanghai, China; ^4^ College of Art and Science, University of San Francisco, San Francisco, CA, USA

**Keywords:** aging, mesenchymal stem cells, recruitment, liver injury, Gerotarget

## Abstract

Mesenchymal stem cells (MSCs) repair tissue injury and may be used to treat immune associated diseases. In carbon tetrachloride (CCl4)-induced liver injury murine model, we administered MSCs. When MSCs were transmitted to young and old mice with liver injury, more MSCs were recruited in old mice. In old mice, inflammation, characterized by TNF-α and IL-6, was increased due to hyper-activation and hyper-function of Kupffer cells. Blocking Kupffer cells decreased MSCs migration in old mice. *In vitro*, Kupffer cells isolated from old mice secreted more inflammatory cytokines and chemokines. Thus, hyper-activation of Kupffer cells in old mice increased recruitment of MSCs after their therapeutic administration.

## INTRODUCTION

Mesenchymal stem cells(MSCs, also called as mesenchymal stromal cells) are a subset of non-hematopoietic adult stem cells which originate from mesoderm. They possess self-renewal ability and pluripotency of differentiation [1-5]. With the growing enthusiasm of stem cell therapy, MSCs have become the most promising candidates for treatment in recent years because their manipulation is free of ethical concerns, without the risk of teratoma formation and with low immunogenicity. In addition, MSCs have been isolated from various tissues successfully such as bone marrow, adipose tissue, umbilical cord, fetal liver, muscle, and lung and can be cultured easily *in vitro* [6-10]. MSCs have been demonstrated to contribute to injury repair, treating degenerative disease and immune related diseases [11, 12]. Increasing clinical trials of MSCs have been applied to the treatment of various diseases since 2004 [13]. During treatment with MSCs, researchers generally have been focusing on the intrinsic property of MSCs. Whereas, the peculiarities of recipients receiving MSCs-treatment have not been paid enough attention, such as the age. As reported, the characteristics of patients varied in terms of age [14-17]. MSCs have to migrate to target sites so as to work as a functional curer. The efficiency of MSCs migration *in vivo* plays a crucial role in the therapeutic effect of MSCs. It has been shown that with higher passage number, the engraftment capacity of MSCs decreased [18]. But whether the age of recipients involves in the engraftment of MSCs has not been investigated. In this study, we investigated engraftment of MSCs in young and old mice by using CCl4 induced mouse liver injury model. Data showed that old mice could attract more MSCs to injured liver.

Inflammation has been shown to be a powerful traction of MSCs. MSCs have the capacity of migrating specifically to damage sites with inflammation [19, 20]. This is also the main reason why MSCs can be used to recover tissue injury and mitigate inflammation. Thus, different degrees of inflammation may be the key factor contributing to the diverse engraftment of MSCs between different ages of recipients. Kupffer cells (KCs) are known as a population of liver resident macrophages, constituting nearly 90% of the tissue macrophages. KCs, residing in the lumen of the liver sinusoids, undergo phagocytosis of larger particulates and foreign materials [21]. They were known to play critical roles in initiation, maintenance and outcome of inflammation. KCs release large amount of inflammatory cytokines and chemokines after activation. As respected, we found that old mice suffered from more serous degree of inflammation which was owing to more KCs infiltration prominently. However, the mechanism by which more KCs were activated in old mice remains to be clarified.

Autophagy, an evolutionarily conserved catabolic pathway from yeast to mammals, serves as a major lysosomal degradation pathway of recycling intracellular components and also eliminating damaged macromolecules, including proteins, lipids, and dysfunctional organelles [22]. Autophagy has also been proposed to be a key regulator of inflammation. It has been shown that autophagy deficiency increases inflammation in tumor cells, whereas activating autophagy has the opposite effect [23]. Increasing evidences have shown that autophagy affected inflammation *via* various mechanisms. White's team demonstrated in 2006 that impaired autophagy induces necrosis and thus stimulates the inflammatory response [23]. Massey and his colleagues showed that deficiency in autophagy is involved in Crohn's disease, suggesting a potential role of autophagy in inhibiting inflammation [24]. Thus, we suspected that different activation levels of autophagy probably resulted in different degree of inflammation. In this study, decreased autophagy activation was observed in KCs isolated from old mice. Autophagy interference could reverse inflammation level and MSCs recruitment. Our data suggested that diverse migration efficiency of MSCs in different ages of recipients was dependent on the different degree of inflammation driven by KCs.

## RESULTS

### Old mice recruited more MSCs to injured liver and represented exacerbated inflammation

**Figure 1:**
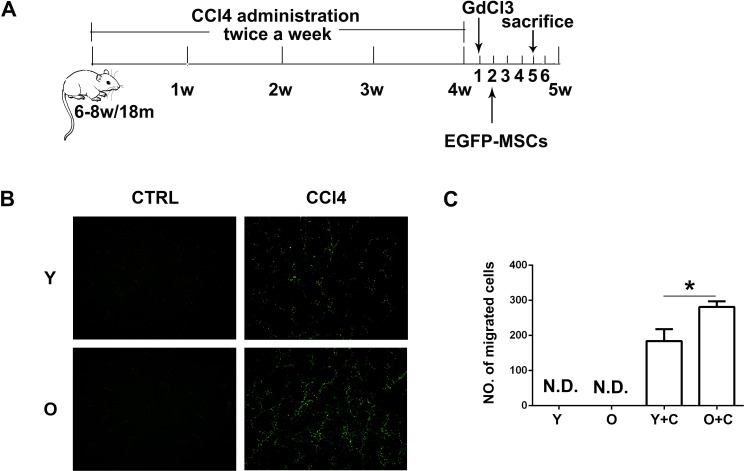
Old mice recruited more MSCs to injured liver **A.**, schematic diagram for mouse liver injury model induced by CCl4. **B.**, Frozen sections of liver were made and MSCs migration to injured liver of indicated groups was detected by fluorescence microscope. (100×) **C.**, The quantification of EGFP-MSCs in **B.**. Y, young; O, old; Y+C, young+CCl4; O+C, old+CCl4. *P < 0.05.

We established a CCl4 induced liver injury model in both young and old mice (Figure 1A). In this model, 6-8-week-old mice were regared as young mice and 18-month-old mice were regarded as old mice. To compare the recruitment of MSCs to both groups of mice, we isolated MSCs from bone marrow of EGFP-transgenic mice and then transplanted them into mice at 5^th^ week after CCl4 treatment. Three days after EGFP-MSCs transplantation, ice sections of mouse liver were made and the results showed there were no MSCs migrating to natural young and old mice. Whereas, in the CCl4 treated groups, more EGFP-MSCs could be observed in the livers of old mice (Figure 1B, 1C).

**Figure 2:**
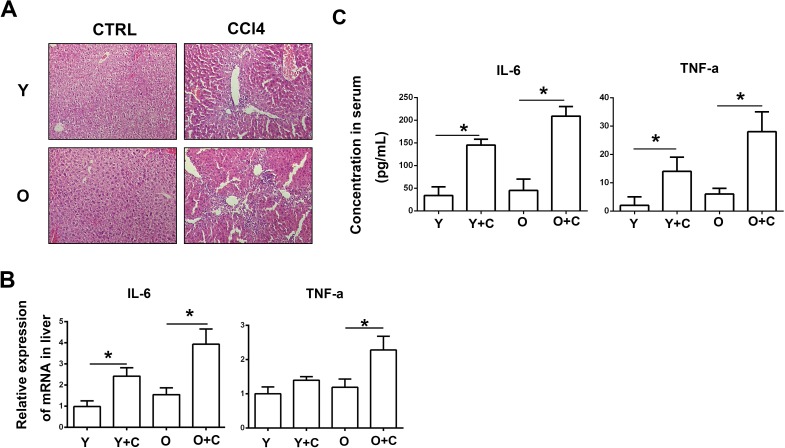
Old mice exhibited exacerbated inflammation after CCl4 administration **A.**, HE staining was performed to detect inflammation level. (200×) **B.**, Concentration of IL-6 and TNF-α in mice serum was detected by ELISA. **C.**, mRNA expression of IL-6 and TNF-α in mice liver was detected by RT-PCR. Y, young; O, old; Y+C, young+CCl4; O+C, old+CCl4. *P < 0.05.

MSCs have the ability to migrate to inflammation and injury sites. Thus, we examined the degree of inflammation among different groups. HE staining results showed that there were more inflammatory cells infiltration in the liver of old mice after intragastric CCl4 infusion (Figure 2A). In addition, we detected two cytokines which can represent inflammation response typically. From Figure 2B and 2C, we can see that expression of TNF-α and IL-6 both in liver and serum was both upregulated in old mice with liver injury. From data above, it can be concluded that old mice exhibited more serious symptoms of inflammation than young mice did after injury.

### More MSCs were recruited to old mice owing to exacerbated degree of inflammation induced by hyper-activation of KCs

**Figure 3:**
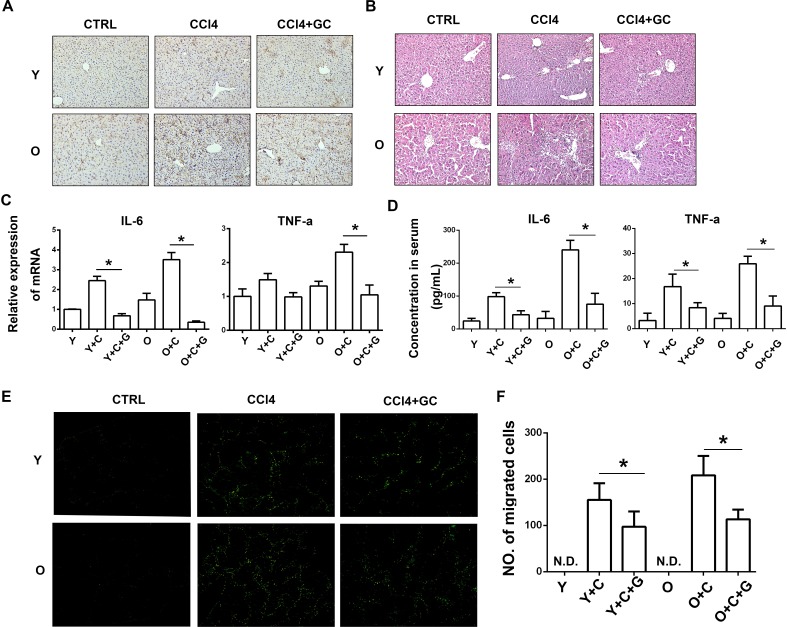
More MSCs were recruited to old mice owing to exacerbated degree of inflammation induced by hyper-activation of KCs **A.**, KCs activation was detected by staining CD68 by immunohistochemistry. (200×) **B.**, HE staining was performed to detect inflammation level. (200×) **C.**, mRNA expression of IL-6 and TNF-α in mice liver was detected by RT-PCR. **D.**, Concentration of IL-6 and TNF-α in mice serum was detected by ELISA. **E.**, Frozen sections of liver were made and MSCs migration to injured liver of indicated groups was detected by fluorescence microscope. (100×) **F.**, The quantification of EGFP-MSCs in **E.**. Y, young; O, old; GC, GdCl3; Y+C, young+CCl4; O+C, old+CCl4. Y+C+G, young+CCl4+GdCl3; O+C+G, old+CCl4+GdCl3. N.D., not detected. *P < 0.05.

It is well known that KCs are resident macrophages in liver sinusoids contributing to host defense and play an important role in inflammation response, especially in response to liver injury [25]. Activated KCs are able to secret a series of inflammatory cytokines such as TNF-α, IL-6 and chemokines [26-28]. To clarify the relationship between inflammation and KCs activation in liver, we detected KCs activation by staining CD68 in liver tissue sections by immunohistochemistry (IHC) assay. As shown in Figure 3A, the IHC results indicated that in CCl4 treated groups, more KCs were activated in old mice, which is consistent with inflammation level. Thus, more inflammation emerged in old mice is probably due to hyper-activation of KCs.

To verify the role of KCs in inflammation and MSCs recruitment, we used Gadolinium chloride (GdCl3, a special inhibitor of KCs) to block the function of KCs. GdCl3 was injected *i.v.* at 24h before MSCs injection. As shown in Figure 3A, CCl4 stimulated activation of KCs significantly in both young and old mice. However, GdCl3 could alleviate the process of KCs activation effectively displayed by decreased CD68 expression. Meanwhile, we use HE staining assay to detect the effect of GdCl3 on inflammation. As a result, GdCl3 treatment also inhibited infusion of other inflammatory cells (Figure 3B). In addition, as shown in Figure 3C, the mRNA expression of IL-6 and TNF-α in fresh liver tissue decreased after GdCl3 administration. The secretion of those two cytokines in serum was detected by ELISA. The ELISA result also showed the same trend (Figure 3D). Collectively, KCs inhibition could suppress inflammation response.

In a further step, the recruitment of MSCs to injured liver *in vivo* was examined after GdCl3 injection. MSCs were injected 24h after GdCl3 administration and the migration was observed through the frozen liver sections three days later. Interestingly, as shown in Figure 3E and 3F, we found that the number of MSCs recruiting to injured liver significantly decreased in CCl4 plus GdCl3 group compared with CCl4 group. Collectively, decreased inflammation induced by blocking KCs activation by GdCl3 resulted in the suppression of MSCs migration.

### KCs isolated from old mice could attract more MSCs *in vitro* due to more inflammatory chemokines secretion

**Figure 4:**
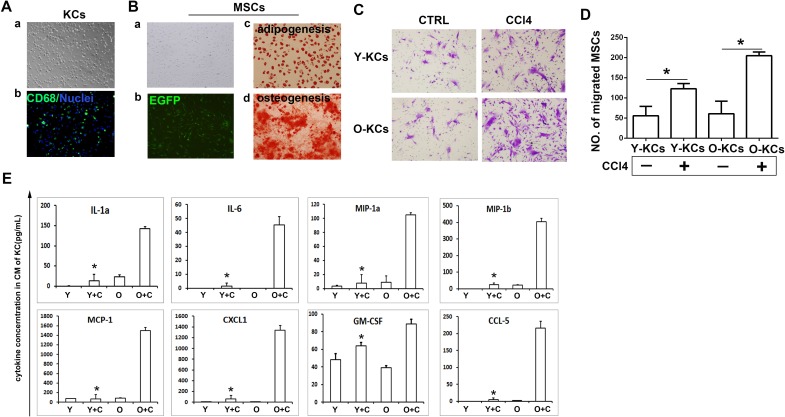
KCs isolated from old mice could attract more MSCs *in vitro* due to more inflammatory chemokines secretion **A.**, Identification of KCs isolated from mice liver. (a**)**Phenotype of KCs isolated from mice liver; (b**)** Cytoimmunofluorescence assay was done to identify KCs(CD68, green; nuclei, blue). (400×) **B.**, Identification of MSCs isolated from mice bone marrow. (a**)**, Phenotype of MSCs; (b**)**, MSCs under fluorescent microscope; (c**)**, Oil red O staining after adipogenesis differentiation; (d**)**, Alizarin Red S staining after osteogenesis differentiation. (200×)**C.**, Transwell migration assay was performed to detect MSCs migration to KCs. Migrated MSCs were stained by crystal violet. (200×) **D.**, The quantification of migrated MSCs of C. **E.**, Cytokines concentration in conditioned medium of KCs was detected by bioplex assay. Y-KCs, KCs from young mice; O-KCs, KCs from old mice; Y, young; O, old; Y+C, young+CCl4; O+C, old+CCl4. *P < 0.05.

As demonstrated above, inflammation induced by KCs activation could promote MSCs migration to injured liver. To discover the underlying mechanism, we isolated KCs from young/old mice with or without CCl4 treatment and detected the function of KCs *in vitro*. We used percoll separating medium to isolate KCs and identified those cells by staining CD68 expression. As shown in Figure 4A, isolated cells appeared to be small and round, and immunofluorescence assay showed that more than 80% of the isolated cells expressed CD68. We also isolated MSCs from bone marrow of normal mice and identified them by adipogenesis and osteogenesis differentiation induction (Figure 4B). Then we used transwell assay to detect the attraction of MSCs by KCs. MSCs were planted on the upper membrane of the transwell, KCs were planted on the lower plate, and MSCs transferred to lower membrane were stained with crystal violet after 72h. The data showed that KCs from normal liver just attracted a few MSCs, while the KCs from liver after CCl4 treatment attracted more MSCs (Figure 4C). From the right panel of Figure 4C, we can see that in both groups with CCl4 treatment, old KCs attracted more MSCs than the young KCs.

To determine the mechanism by which old KCs attracted more MSCs, we detected the cytokines secretion in the conditioned medium of KCs by bioplex assay. As shown in Figure 4D, the data indicated that old KCs from CCl4 treated mice produced more inflammatory cytokines and chemokines. IL-1a and MIP-1a are related to inflammatory directly. MIP-1b, MCP-1, CCL5, CXCL1 could recruit NK cells, T cells, monocytes and other immune cells to exacerbate inflammation. GM-CSF could promote granulocytes and monocytes production. They not only promote inflammation response directly or indirectly, but also are chemokines contribute to MSCs migration [29]. The data indicated that KCs present hyper-function in old mice.

### Autophagy in KCs was impaired in old mice after CCl4 treatment

**Figure 5:**
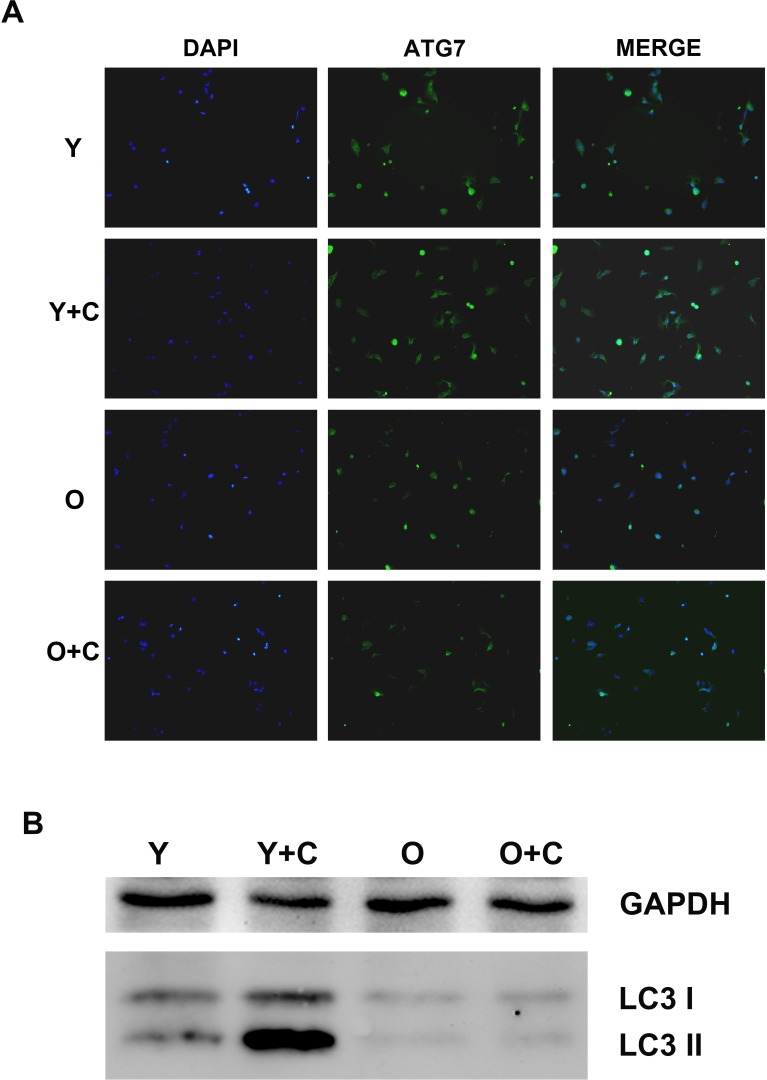
Autophagy in KCs was impaired in old mice after CCl4 treatment **A.** Autophagy activation in KCs was detected by staining ATG7 by immunofluorescence assay. ATG7 was presented by green fluorescence, DAPI was used to stain nuclei(400×). **B.** LC3 I/II was tested by western blotting assay. Y, young; O, old; Y+C, young+CCl4; O+C, old+CCl4.

From both the *in vivo* and *in vitro* data, inflammation is the key driver recruiting MSCs. Nevertheless, what results in the difference of inflammation between young and old mice after CCl4 treatment needs a further study. Autophagy, as an evolutionarily conserved catabolic pathway from yeast to mammals that serves as a major lysosomal degradation pathway for recycling intracellular components, has been proposed to be a key regulator of inflammation *via* various mechanisms. There has also been evidence indicating that autophagy deficiency increases inflammation. To determine whether autophagy play a key role in the increase of inflammation in old mice, we detected autophagy activation in KCs. ATG7, a key regulator of autophagy activation pathway, was detected by immunofluorescence assay, and the data indicated that CCl4 could stimulate autophagy activation significantly in young KCs, but on the contrary, there was no obvious increase of autophagy activation could be observed in old KCs (Figure 5A). In addition, we examined microtubule-associated protein 1 light chain 3 (LC3) expression in KCs, which could transform from type I (LC3I)to type II (LC3II) when autophagy was induced. As shown in Figure 5B, The western blotting result showed that LC3I and LC3II increased significantly in KCs from young mice after CCl4 treatment. Meanwhile, in the two groups of old KCs, LC3I and LC3II both expressed slightly. All of these results indicated that autophagy in old KCs was impaired compared with young KCs.

### Autophagy interference reversed recruitment of MSCs to KCs

**Figure 6:**
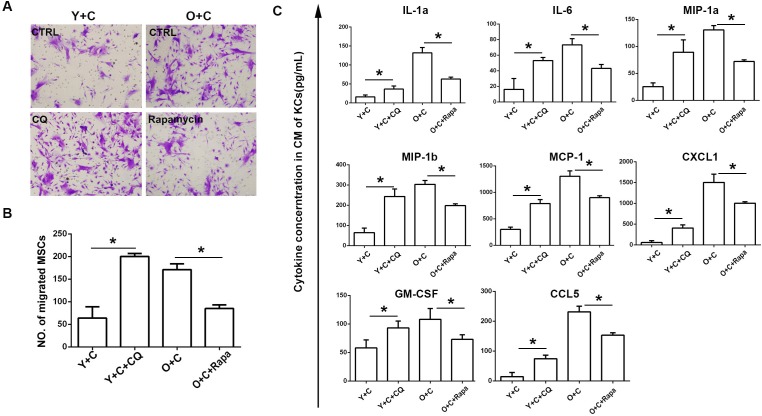
Autophagy interference reversed recruitment of MSCs to KCs **A.**, Transwell migration assay was performed to detect MSCs migration to KCs after autophagy interference. Migrated MSCs were stained by crystal violet(200×). KCs from Y+C mice were treated with 5μM CQ; KCs from O+C mice were treated with 100nm rapamycin. **B.** The quantification of migrated MSCs of A. **C.**, Cytokines concentration in conditioned medium of KCs after autophagy interference was detected by bioplex assay. Y+C, young+CCl4; Y+C+CQ, young+CCl4+CQ; O+C, old+CCl4; O+C+rapamycin, old+CCl4+rapamycin. *P < 0.05.

Based on data above, we have demonstrated that autophagy could not be induced effectively by CCl4 in old KCs. To verify autophagy is the key reason involved in MSCs recruitment, we interfered autophagy in KCs by using autophagy inhibitor CQ and autophagy stimulator rapamycin. We used CQ to inhibit autophagy in young KCs and used rapamycin to induce autophagy in old KCs, and then examined the effect of autophagy interference on MSCs recruitment. As shown in Figure 6A, compared with control, CQ promoted recruitment of MSCs to young KCs. For old KCs, rapamycin suppressed the recruitment of MSCs. These data suggested that autophagy in KCs may be the main reason which determines the ability of recruiting MSCs. In addition, we tested the inflammatory cytokines alteration after inhibiting or inducing autophagy in different groups of KCs. We collected conditioned medium of KCs in different groups and tested a series of cytokines in them by bioplex assay. The results indicated that autophagy inhibition could increase inflammation and autophagy stimulation could decrease inflammation instead (Figure 6B).

## DISCUSSION

MSCs have been demonstrated to have powerful potential in repairing injury and treating immunity related diseases. Although the “gold rush” to use MSCs in clinical settings began with high enthusiasm in many countries, with China, Europe and US leading the way (http://clinicaltrial.cn), numerous scientific issues remain to be resolved before the establishment of clinical standards and governmental regulations. During cell therapy, to get better treatment effect, individualized treatment is very important and necessary apart from considering the property of employed cells. Among the big patients cohort, age is an important factor which deserves consideration. Among different ages, people always present a big difference during treatment.

During cell therapy, the migration of cells to targeted tissues is a pivotal step and the migration efficiency determines the treatment effect to some extent. To study the cell migration efficiency in young and old individuals, we established liver injury model by CCl4 administration in young and old mice. Exogenous MSCs isolated from bone marrow were administered following the liver injury model. We found that under the same dosage of CCl4 treatment, there were more MSCs recruiting to injured liver in old mice. This result indicated that liver injury microenvironment varies between young and old mice. The different capacity of recruiting MSCs determines that different therapeutic regimens should be used to different ages. On the other hand, our result may support the fact that old people tend to have more fibrosis following tissue injury because MSCs have the potential to differentiating into myofibroblasts which produce collagen.

In a further step, we found that more MSCs recruiting to old mice was dependent on more inflammation activation. In the old mice liver, we could see more inflammatory cells invasion and inflammatory cytokines expression. This result is consistent with the idea “Chronic inflammation is associated with aging and age-related diseases” [30, 31]. In addition, we found that in liver injury, there have been more KCs activated in old mice than in young mice. KCs are important immune cells involved in inflammation. When KCs were blocked, inflammation and MSCs engraftment were both reduced. We also found that KCs isolated from old mice liver with injury could secret more inflammatory cytokines and chemokines. These data indicated that the hyperfunction of KCs in old mice was the key reason driving more inflammation accumulation and MSCs recruitment. Overstimulation of KCs in old mice is probably due to the feedback block of inflammation regulation signaling pathways. In previous studies, cellular hyperfunction with aging has been demonstrated. Mikhail V. raised the question aging maybe caused by hyperfunction of cells instead of accumulation of molecular damage [32]. And this hyperfunction theory was suitable to explain aging in *C. elegans* [33]. However, aging was also considered to resulting from energy excess [34].

Autophagy, as an important process to keep homeostasis, was reported to have the ability to downregulate inflammation. Lower autophagy activation level was found in old KCs. Autophagy interference in KCs could reverse chemokines production and MSCs migration. Thus, our results suggested that with aging, hyperfunction of KCs was probably owing to deficiency of autophagy. The big difference of autophagy activation is probably a main reason resulting in homeostasis diversity between young and old ones.

Taken together, our study indicated that old recipients could recruit more MSCs to injury sites due to more inflammation induced by hyperfunction of KCs. And it is the autophagy activation defection that resulted in the hyperfunction of old KCs. Thus, our results suggested that cell therapy needs individual therapy, and the age should be considered to be an important parameter. Therapeutic regimens, especially the dosage of MSCs, needs to be reconsidered upon different ages. However, the subsequent function and behaviours of MSCs after locating in targeted tissues or organs needs a further investigation.

## MATERIALS AND METHODS

### Materials

Dulbecco's modified eagle medium(low glucose), RPMI 1640 medium, fetal bovine serum(FBS), glutamax(Catalog number: 35050-061), penicillin-streptomycin(Catalog number:15140-122), trypsin-EDTA(Catalog number: 15400-054) and basic fibroblast growth factor (bFGF) (Catalog number: PMG0035), PMSF Protease Inhibitor (Catalog nuber: 36978), Alexa Fluor 488 goat anti-mouse IgG secondary antibody (Catalog: R37120), DAPI (Catalog: D1306) were purchased from Thermo Fisher Scientific, Inc.. Anti-GAPDH antibody(Catalog number: ab181602) was purchased from Abcam, Inc.. Anti-LC3 antibody(Catalog number: 4108) and anti-rabbit IgG, HRP-linked antibody(Catalog number: 7074) was purchased from Cell Signaling Technology, Inc.. Anit-ATG7 antibody(Catalog number: MAB6608) was purchased from R&D system, Inc.. Bio-Plex cytokine array system (Catalog number: 3156) was purchased from Bio-Rad Laboratories, Inc.. Type IV collagenase(Catalog number: C5318) was purchased from Sigma-Aldrich Co. LLC. Percoll(Catalog number: 17-0891-01) was purchased from Pharmacia, Inc..

### Isolation and culture of mouse MSCs

Enhanced green fluorescence protein (EGFP) transgenic or WT male C57/BL6 mice aged 6-8 weeks were used to isolate bone marrow MSCs. MSCs were isolated and cultured as described by Heng Zhu, et al. [35]. Mice were killed by cervical dislocation and sterilized by 75% ethanol for 5 min. Then the limbs were removed. We flushed bone marrow cells from the medullary cavities from the tibias and femurs with physiological saline by using a 2 mL needle. Derived bone marrow cells were suspended in single cell and seeded in DMEM medium(low glucose) with 10%FBS, 1×glutamax, 1×penicillin-streptomycin by 1×10^7^ cells/mL. Three days later, suspended cells were removed and medium was replaced with fresh medium, the adherent cells stayed. Thereafter, medium was replaced every three days. At the seventh day after isolation, attached cells were removed by trypsin-EDTA, then resuspended in fresh medium. After cells were passaged for 3 times, they were taken as purified MSCs and identified by adipocytes and osteoblasts differentiation referred to our former work [36]. In the following culture of MSCs, bFGF was added at a concentration of 0.5ng/mL.

### Isolation and culture of mouse KCs

Mouse KCs were separated and cultured by type IV collagenase digestion method modified based on Peizhi Li's protocol [37]. Mice after different treatment were anaesthetized by 4% chloral hydrate intraperitoneal injection at 100μL/10g weight. The portal vein was inserted with a plastic catheter (outside diameter 0.6mm) and the liver was perfused with 20mL Ca^2+^ -Mg^2+^ -free HBSS (3mL/min), with post cava clipping to drive out red cells. Thereafter, liver was perfused with 5 mL 0.1% type IV collagenase and resected. The resected liver was minced into small pieces as much as possible in a 35 mm culture dish and then transferred into a 50 mL centrifuge tube with another 3mL collagenase. Then the liver tissue was bathe-watered at 37°C for 30 min, mixed gently per 10 min. Following digestion, the liver homogenate was filtered with a 70μm strainer to remove the undigested tissue and the strainer was flushed with 1640 medium containing 10% FBS. The cell suspension was collected and centrifuged at 600rpm for 3min to remove liver parenchymal cells. The supernatant was transferred to a new 10mL centrifuge tube and centrifuged at 1500rpm for 5min. The supernatant was discarded and the cell sediments mainly contained KCs, HSCs and sinusoidal endothelial cells. To purify KCs from above non-parenchymal cells, we used percoll discontinuous gradient separation method. At the bottom of the 10mL centrifuge tube was 4mL 60% percoll, in the middle is 4mL 30% percoll, and at the top of the tube is 4mL HBSS containing the cell sediments collected above. Every layer should be added carefully and slowly to avoid mixing with each other. This compound with 3 layers was centrifuged at 2000rpm for 15min and the liquid came to 4 layers. The top and the bottom layer were discarded, and the middle two layers were collected to a new 10mL layer followed by centrifugation at 1500rpm for 5min. The sediments were suspended with 1640 medium containing 10% FBS and seeded in 6-well plates at density of 1-3×10^7^ per well. 2 hours later, non-adherent cells were removed and new 1640 medium with 10% FBS was added. The adherent cells were used as KCs.

KCs were identified by immunofluorescence using anti-CD68 antibody.

### Mouse model

In order to establish chronic liver injury, male C57BL/6 mice aged 6-8 weeks(young) and 18 months(old) were feeded with 20%(v/v, dissolved in olive oil) carbon tetra-chloride(CCl_4_) by intragastric method at a concentration of 5.0mL/kg, twice a week. In our model, gadolinium chloride(GdCl3, GC) was used to block KCs *in vivo*. At the fifth week, young and old mice were divided into two groups separately, including control and CCl4 group, which were named as Y(young) group, Y+C(young+CCl4) group, O(old) group and O+C(old+CCl4) group. EGFP-MSCs(1×10^6^ per mouse) were transplanted to all the mice through the tail vein. Furthermore, Y+C+GC(young+CCl4+GdCl3) and O+C+GC(old+CCl4+GdCl3) group were added. In these two groups, 2mg/mL GC was injected i.v. at a concentration of 100μL/20g one day before MSCs injection. At the fourth day after MSCs injection, all the mice were sacrificed and liver tissue and serum were collected.

### Transwell migration assay

KCs were separated from four groups of mouse liver, including young, young+CCl4, old, old+CCl4. To examine the effect of KCs recruiting MSCs, we seeded KCs in 24-well plate at a density of 5×10^4^ per well with 1640 medium containing 5% FBS, 2×10^4^ MSCs were plated in the transwell insert(8μm pore) with serum-free DMEM medium. After 72h, the upper cells in the inserts were wiped by cotton swab, cells transferred to the lower surface of the inserts were stained with 0.1% crystal violet solution for 1 hour. In the further test, to detect the role of autophagy in recruiting MSCs, KCs from Y+C mice were treated with 5μM chloroquine(CQ), KCs from O+C mice were treated with 100nm rapamycin at the same time of coculture of KCs and MSCs.

### Immunohistochemistry and immunofluorescence staining

Paraffin sections used for immunohistochemistry assay was 5-μm thick, and the detail process is done according to method described before [38]. Antibodies employed in our experiment, anti-CD68 and anti-ATG7 were diluted by 1/200 for using. Alexa Fluor 488 goat anti-mouse IgG secondary antibody was used at a concentration of 1/200 correspondingly. Nucleus were counter stained with DAPI at 1μg/mL.

### Real-time PCR

Total RNA from freezed liver tissues were extracted by trizol method. 1μg RNA in 10 μL for all reagents was transcripted to cDNA using bestar qPCR RT kit. The real-time PCR was performed using bestar real time PCR master mix with an ABI Prism 7300 system. The primers for real-time PCR were as follows: TNF-α, sense,5′-TTCTGTCTACTGAACTTCGGGGTGATCGGTCC-3′, and antisense, -GTATGAGATAGCAAATCGGCTGACGGTGTGGG-3′; IL-6, sense, 5′-GAGGATACCACTCCCAACAGACC-3′, and antisense, 5′-AAGTGCATCATCGTTGTTCATACA-3′; GAPDH, sense, 5′-AGGTCGGTGTGAACGGATTTG-3′, and antisense, 5′-TGTAGACCATGTAGTTGAGGTCA-3′.

### Cytokine detection assay

Concentrations of TNF-α and IL-6 in mouse serum were assayed by ELISA kit according to the manufacturer's recommendation. Concentrations of IL-1α, IL-6, MIP-1α, MIP-1β, MCP-1, CXCL1, GM-CSF and CCL5 were measured in conditioned medium of KCs using the Bio-Plex cytokine array system (Bio-Rad) according to the manufacturer's instructions. KCs isolated from mouse liver were cultured with serum-free 1640 medium for 24h, then supernatants were collected as conditioned medium.

### Haematoxylin and eosin(HE) staining

Mouse liver samples were obtained after sacrifice and fixed in 4% paraformaldehyde, and then embedded in paraffin. 5-μm-thick sections were prepared for the experiments. Mouse liver sections embedded in paraffin were stained with haematoxylin and eosin according to the manufacture's protocol.

### Western blotting assay

KCs treated with appropriate conditions were lysed in lysis buffer. The lysates were qualified using BCA kit and boiled with loading buffer for 10 minutes. Thereafter, denatured protein was segregated by SDS-polyacrylamide gel electrophoresis(SDS-PAGE), and then transferred onto nitrocellulose(NC) membrane. The membranes were blocked by 5% nonfat milk, and then immunoblotted by various primary monoclonal antibodies. Anti-GAPDH was used at 1/10000 dilution, LC3 was used at 1/1000 dilution. The horseradish peroxidase(HRP)-conjugated goat anti-rabbit secondary antibody and ECL were used to examine the expression of protein. GAPDH was used as internal conference. Expression of target proteins were normalized to GAPDH before comparing among different groups.

### Statistical analyses

All experiments were performed in triplicate at least. Results were presented as means±S.E. and the statistical differences were analyzed by student's *t*-test, *n* = 3 (*p* < 0.05 was considered significant).

## Abbreviations

MSCs: mesenchymal stem cells
CCl4: carbon tetrachloride
TNF-α: tumor necrotic factor
IL-6: interleukin-6
KCs: Kupffer cells
ATG7: Autophagy-related protein 7
LC3: microtubule-associated protein 1 light chain 3
GdCl3: Gadolinium chloride
MIP-1β: Macrophage Inflammatory Protein-1β
MCP-1: monocyte chemotactic protein 1
CCL5: (C-C motif) ligand 5
CXCL1: (C-X-C motif) ligand 1
GM-CSF: granulocyte macrophage-colony stimulating factor
CQ: chloroquine
