# Correction: Kupffer cells-dependent inflammation in the injured liver increases recruitment of mesenchymal stem cells in aging mice

**DOI:** 10.18632/oncotarget.27781

**Published:** 2020-10-20

**Authors:** Xue Yang, Lei Liang, Chen Zong, Fobao Lai, Pengxi Zhu, Yu Liu, Jinghua Jiang, Yang Yang, Lu Gao, Fei Ye, Qiudong Zhao, Rong Li, Zhipeng Han, Lixin Wei

**Affiliations:** ^1^ Tumor Immunology and Gene Therapy Center, Eastern Hepatobiliary Surgery Hospital, the Second Military Medical University, Shanghai, China; ^2^ Medical College of Soochow University, Suzhou, China; ^3^ Department of Pharmacy, Chang Hai Hospital, the Second Military Medical University, Shanghai, China; ^4^ College of Art and Science, University of San Francisco, San Francisco, CA, USA

**This article has been corrected:** Due to errors during figure assembly, the wrong transwell image was used for the lower left panel of Figure 6A - it is an accidental duplicate of the lower right panel of Figure 4C. The corrected Figure 6, obtained using original data, is shown below. The authors declare that these corrections do not change the results or conclusions of this paper.

Original article: Oncotarget. 2016; 7:1084–1095. 1084-1095. https://doi.org/10.18632/oncotarget.6744

**Figure 6:**
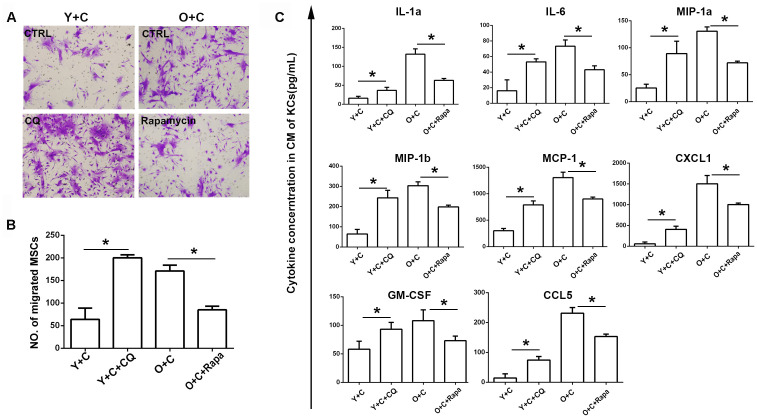
Autophagy interference reversed recruitment of MSCs to KCs. (**A**) Transwell migration assay was performed to detect MSCs migration to KCs after autophagy interference. Migrated MSCs were stained by crystal violet(200×). KCs from Y+C mice were treated with 5μM CQ; KCs from O+C mice were treated with 100nm rapamycin. (**B**) The quantification of migrated MSCs of A. (**C**) Cytokines concentration in conditioned medium of KCs after autophagy interference was detected by bioplex assay. Y+C, young+CCl4; Y+C+CQ, young+CCl4+CQ; O+C, old+CCl4; O+C+rapamycin, old+CCl4+rapamycin. ^*^ *P* < 0.05.

